# Caution Is Necessary for Acceptance of Motor Units With Intermediate Matching in Surface EMG Decomposition

**DOI:** 10.3389/fnins.2022.876659

**Published:** 2022-05-26

**Authors:** Maoqi Chen, Ping Zhou

**Affiliations:** Biomedical and Rehabilitation Engineering Program, University of Health and Rehabilitation Sciences, Qingdao, China

**Keywords:** surface EMG, motor unit, EMG decomposition, blind source separation, performance validation

## Introduction

Significant progress has been achieved in decomposition of surface electromyographic (EMG) signals, particularly with advances in surface electrode array recording and processing techniques (Holobar and Zazula, 2007; Chen and Zhou, 2016; Negro et al., 2016, among others). In this development, a key issue is how to validate the decomposition yield. Various approaches have been proposed, including EMG simulation (Dai and Hu, 2019; Mohebian et al., 2019), two-source validation (Mambrito and De Luca, 1984; Holobar et al., 2010; Marateb et al., 2011; Negro et al., 2016; Chen et al., 2018a), and decompose-synthesize-decompose-comparisons test (Nawab et al., 2010). The agreement in spike trains between the decomposed and reference motor units is often used to evaluate the performance of surface EMG decomposition algorithms. For example, “two-source” validation involves comparing the decomposition results of simultaneously collected surface and intramuscular EMG signals. The agreement in discharge timing of the common motor units from both types of recordings can be viewed as a performance index of the surface EMG decomposition, given that intramuscular EMG decomposition has been well established (Parsaei et al., 2010).

## Motor Units With Intermediate Matching Require Attention

**Figure 1:**
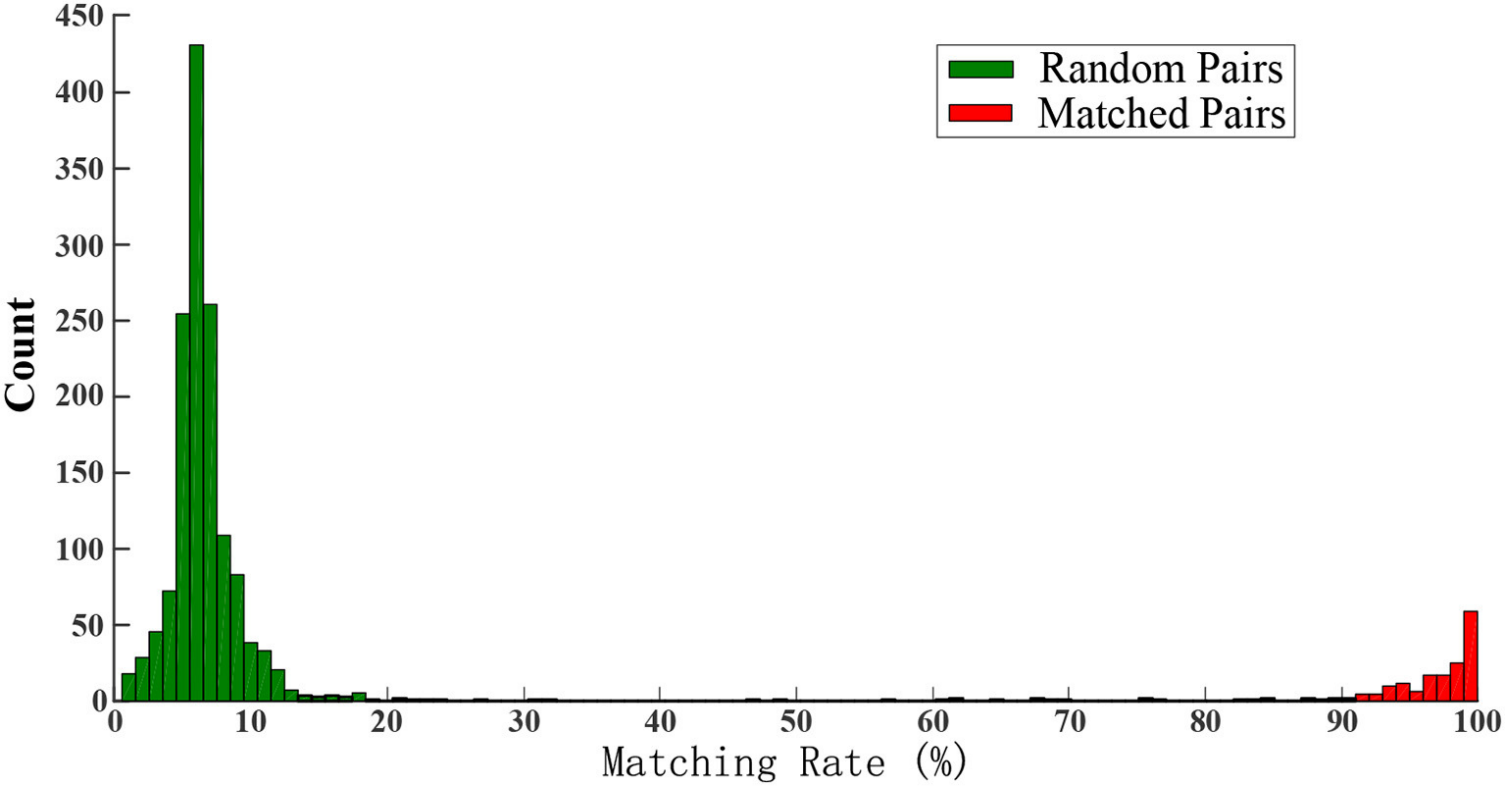
Distribution of *MR* between random pairs of surface and intramuscular motor unit spike trains in individual trials. The matched pairs are shown in red bars (from Chen et al., 2018a, with copyright permission).

Two measures have been used to assess the degree of matching of two spike trains, including matching rate (MR) (Chen et al., 2018a) and rate of agreement (RoA) (Holobar et al., 2010), defined as:


(1) $$\begin{array}{r} MR=\frac{2C}{A+B}\cdot 100\% \end{array}$$

(2) $$\begin{array}{r} RoA=\frac{C}{A+B-C}\cdot 100\% \end{array}$$

where *A* and *B* are the total number of spikes of the two motor unit spike trains, respectively, and *C* is the number of common spikes. *MR* is indeed a F1-score measure if one of the spike trains is deemed as the ground truth, which accounts for both precision and recall. If two motor units match well, their *MR* or *RoA* will be close to 1. For two random motor units, the *MR* or *RoA* will be close to 0. Such a bimodal distribution has been observed in validation studies of EMG decomposition, where there are two peaks (close to 1 or 0) with unambiguous interpretation. For example, in a two-source test performed by Chen et al. (2018a), a distinct bimodal distribution of *MR* was observed with few intermediate values (Figure 1). Note that a high threshold (0.8) of *MR* was used to judge whether two spike trains are from a common motor unit or not, which restricts the number of common motor units but favors a high *MR*. It is notable that different or lower thresholds were used by others in the search for common motor units, resulting in larger numbers of common motor units at the expense of matching degree. For example, two spike trains were considered as from a common motor unit if the number of their matching spikes reached 30 or 50% of the total number of spikes (i.e., *C* ≥ 30%*A* and *C* ≥ 30%*B*; or *C* ≥ 50%*A* and *C* ≥ 50%*B*) (Negro et al., 2016; Dai and Hu, 2019). This corresponds to *MR* ≥ 30% (or 50%) and *RoA* ≥ 17.6% (or 33.3%). It is therefore not surprising to observe a few motor units with intermediate matching in subsequent common motor unit evaluation. For example, *RoA* as low as 45% was reported in a two-source test (Negro et al., 2016), suggesting that this specific motor unit was not completely decomposed. This raises the issue of how to identify and exclude less confident motor units (i.e., those with intermediate *RoA* or *MR*) in routine experiment surface EMG decomposition.

## Examination of Intermediate Matching in Experimental Surface Emg Decomposition

In routine experimental surface EMG decomposition, no reference motor unit spike trains are available for calculation of the level or extent of matching, whereas simulation or simultaneous intramuscular and surface EMG recordings incorporate reference trains. However, examinations can still be carried out toward examining motor units that have intermediate matching. One approach is to calculate *MR* or *RoA* values among all extracted motor units *via* surface EMG decomposition. The other approach is to decompose the same surface EMG signal repeatedly but independently, and then calculate the *MR* or *RoA* of the extracted motor units from two decompositions.

For either of the two proposed approaches, if two spike trains have intermediate *MR* or *RoA*, the chance of their originating from two different motor units is very low, given that the *MR* of a random pair of spike trains is usually < 20% (the longer the length of spike train, the lower the *MR*). Therefore, two spike trains with intermediate *MR* or *RoA* values are most likely from the same motor unit, with at least one spike train having considerable missing spikes (false negatives), or erroneous spikes (false positives), or both. Conceivably, they may reflect duplicate motor units, with inconsistent or unstable performance (i.e., the algorithm does not always converge to a consistent result).

In fact, most surface EMG decomposition algorithms based on blind source separation (BSS) use a parallel search strategy, i.e., they repeatedly search for a number of motor units from the original signal and then deal with duplicates at the end. A usual processing method is to keep the spike train with the highest silhouette (SIL) value among the duplicates (Dai and Hu, 2019). However, solely relying on SIL is not sufficient to exclude those motor units with intermediate matching. As long as the decomposition algorithm converges to the same motor unit, the result should be consistent. Intermediate *MR* or *RoA* indicates that the decomposed motor unit may not be repeatable or stable. Therefore, in routine experimental surface EMG decomposition, the matching index should also be examined to assure the validity of every decomposed motor unit.

## Strategies To Limit the Number of Motor Units With Intermediate Matching

Most BSS-based methods (including FastICA) tend to converge to duplicate motor units. How to overcome this limitation, especially for units with intermediate matching, should be considered in surface EMG decomposition. For example, the progressive FastICA peel-off (PFP) framework (Chen and Zhou, 2016) introduces a “peel off” strategy to mitigate the effects of the already identified motor units on the FastICA convergence, which can reduce the chance of duplicate motor units and therefore more motor units can emerge. To avoid possible cumulative error caused by the progressive peel-off, a series of signal processing techniques are designed to ensure the accuracy of the subtracted spike trains (Chen et al., 2018b). These include valley-seeking clustering to process FastICA output to highlight corresponding motor unit spikes, constrained FastICA to assess extracted spike trains and correct possible erroneous or missed spikes, and several reliability judgments of the spike trains before final acceptance as a valid result. These procedures collectively guarantee the reliability and stability of the extracted motor unit spike trains. As a result, few motor units with intermediate *MR* can be observed (Figure 1).

However, if some of the preset restrictions are relaxed, the BSS based surface EMG decomposition is likely to extract more motor units with decreased or intermediate matching. This highlights the importance of examining some type of matching index. For motor units with intermediate *MR* or *RoA*, one recommendation is to apply a constrained FastICA processing. A reliable spike train is supposed to drive FastICA of the original EMG signals to converge toward an independent component corresponding to itself if it is used as a temporal constraint. During this process, possible erroneous or missed spikes are likely corrected by constrained FastICA.

## Concluding Remarks

In recent decades, different surface EMG decomposition methods, programs and software have been developed and become available to research community. In practical applications, potential users (particularly novice or lay users) may likely take all the program outputs as valid motor units for further analysis. However, although most decomposed outputs can be reliable, there is still a risk of occurrence of a few less reliable motor units. This opinion article is not meant to perform a comprehensive and thorough analysis of these motor units. Rather, it serves as an alert or a reminder to potential users on possible occurrence of less reliable motor units. In particular, intermediate matching of two spike trains may suggest an origin from duplicate motor units with inconsistent or unstable decomposition performance. Therefore, caution is required for acceptance of motor units with intermediate *MR* or *RoA* in surface EMG decomposition.

## Author Contributions

MC wrote the first draft of the manuscript. PZ revised the manuscript. Both authors contributed to the conception and design of the work and approved the submitted version.

## Funding

This article was supported by the Shandong Provincial Natural Science Foundation under Grant Nos. ZR2021QH053 and ZR2020KF012.

## Conflict of Interest

The authors declare that the research was conducted in the absence of any commercial or financial relationships that could be construed as a potential conflict of interest.

## Publisher's Note

All claims expressed in this article are solely those of the authors and do not necessarily represent those of their affiliated organizations, or those of the publisher, the editors and the reviewers. Any product that may be evaluated in this article, or claim that may be made by its manufacturer, is not guaranteed or endorsed by the publisher.
